# Gliotoxin Inhibits Proliferation and Induces Apoptosis in Colorectal Cancer Cells

**DOI:** 10.3390/md13106259

**Published:** 2015-10-02

**Authors:** Junxiong Chen, Chenliang Wang, Wenjian Lan, Chunying Huang, Mengmeng Lin, Zhongyang Wang, Wanling Liang, Aikichi Iwamoto, Xiangling Yang, Huanliang Liu

**Affiliations:** 1 Guangdong Institute of Gastroenterology and the Sixth Affiliated Hospital, Sun Yat-sen University, Guangzhou 510655, Guangdong, China; E-Mails: junxiong_ch@sina.com (J.C.); chenliangwang1984@gmail.com (C.W.); huangchunyingdg@163.com (C.H.); lmmhello@163.com (M.L.); seyyawang@163.com (Z.W.); 2 Guangdong Provincial Key Laboratory of Colorectal and Pelvic Floor Diseases, Sun Yat-sen University, Guangzhou 510655, Guangdong, China; 3 Institute of Human Virology and Key Laboratory of Tropical Disease Control of Ministry of Education, Zhongshan School of Medicine, Sun Yat-sen University, Guangzhou 510080, Guangdong, China; 4 School of Pharmaceutical Sciences, Sun Yat-sen University, Guangzhou 510006, Guangdong, China; E-Mails: lanwj@mail.sysu.edu.cn (W.L.); natprodlwl@gmail.com (W.L.); 5 Advanced Clinical Research Center, Institute of Medical Science, University of Tokyo, Tokyo 108-8639, Japan; E-Mail: aikichi@zj9.so-net.ne.jp

**Keywords:** gliotoxin, marine secondary metabolites, anti-tumor activity, Wnt signaling pathway, colorectal cancer, proliferation, apoptosis

## Abstract

The discovery of new bioactive compounds from marine natural sources is very important in pharmacological research. Here we developed a Wnt responsive luciferase reporter assay to screen small molecule inhibitors of cancer associated constitutive Wnt signaling pathway. We identified that gliotoxin (GTX) and some of its analogues, the secondary metabolites from marine fungus *Neosartorya pseufofischeri*, acted as inhibitors of the Wnt signaling pathway. In addition, we found that GTX downregulated the β-catenin levels in colorectal cancer cells with inactivating mutations of adenomatous polyposis coli (APC) or activating mutations of β-catenin. Furthermore, we demonstrated that GTX induced growth inhibition and apoptosis in multiple colorectal cancer cell lines with mutations of the Wnt signaling pathway. Together, we illustrated a practical approach to identify small-molecule inhibitors of the Wnt signaling pathway and our study indicated that GTX has therapeutic potential for the prevention or treatment of Wnt dependent cancers and other Wnt related diseases.

## 1. Introduction

Inappropriate activation of Wnt signaling has been observed in numerous human cancers [1,2]. The hallmark of this pathway is that it activates the transcriptional role of β-catenin. In canonical Wnt signaling, Wnt ligands binding to a complex comprised with Frizzled and Lrp5/6 receptors lead to the inactivation of the destruction complex, which was formed with proteins including Axin, adenomatous polysis coli (APC), and glycogen synthase kinase 3β (GSK-3β), leading subsequently to the stabilization of β-catenin and its translocation to the nucleus [3]. In the nucleus, β-catenin promotes the transcription of Wnt target genes through partnerships with the T cell-specific transcription factor/lymph enhancer-binding factor 1 (TCF/LEF) family of transcription factors [3]. Notably, the Wnt pathway is known to be altered in over 90% of colorectal cancers, including inactivating mutations of APC or activating mutations of β-catenin [4,5,6]. APC is an integral part of the destruction complex that controls cytoplasmic β-catenin levels by promoting ubiquitin-mediated degradation of β-catenin. Cancer associated mutations in APC, thus lead to an excessive accumulation of β-catenin and the concomitant expression of Wnt/β-catenin targeting genes, which together play important roles in colorectal tumorigenesis and metastasis. Activating mutations in β-catenin that abrogate its regulation by APC represent an alternative route to Wnt activation [7,8]. Mutations in SOX9 (which facilitates the degradation of β-catenin) [9], TCF7L2 (the Wnt signaling effector), DKK1 (dickkopf Wnt signaling pathway inhibitor 1) family members, AXIN2 (an integral member of the destruction complex and a transcriptional downstream target of canonical Wnt signaling), FBXW7 (F-box and WD repeat domain containing 7, E3 ubiquitin protein ligase and is responsible for Wnt induced target genes degradation), FAM123B (an X-linked negative regulator of Wnt signaling), and ARID1A (a member of the SWI/SNF family and is also involved in the regulation of Wnt signaling) [10]. Meanwhile, the extremely high levels of over-expression of the Wnt ligand receptor gene FZD10 has also been reported in CRC [4]. Altogether, these altered Wnt pathway genes reveal the critical role of Wnt in CRC. Given that the majority of colorectal cancers are involved activation of the Wnt signaling pathway, and multiple mutations lead to this activation, developing drugs which target the Wnt signaling, particularly β-catenin, is an attractive strategy for CRC treatment.

Marine-derived fungi has been proven to be a prolific source of secondary metabolites with interesting structural properties and biological activities [11,12,13]. For example, Plinabulin (NPI-2358), a natural product isolated from a marine alga associated *Aspergillus* sp. Plinabulin is shown to be a vascular disrupting agent that elicits tumor vascular endothelial architectural destabilization leading to selective collapse of established tumor vasculature and is currently under clinical trials [14,15]. In recent years, we have optimized cultivation parameters to isolate and identify a series of novel and/or bioactive metabolites form marine fungi [16,17,18,19]. GTX and some of its analogues, the secondary metabolites of marine fungus *Neosartorya pseudofischeri*, have previously been shown to exert potential cytotoxic effects on CRC cell lines [19].

In this study, we developed a Wnt responsive luciferase reporter assay to screen small molecule inhibitors of cancer associated constitutive Wnt/β-catenin signaling pathway and identified gliotoxin (GTX) and some of its analogues as small molecule Wnt inhibitors. GTX blocked the Wnt/β-catenin signaling pathway and repressed the expression of β-catenin dependent genes. In addition, GTX decreased the β-catenin levels in CRC cells with inactivating mutations of APC or activating mutations of β-catenin. Furthermore, we demonstrated that GTX induced growth inhibition and apoptosis in several CRC cell lines. Together, we illustrated a practical approach to identify small molecule inhibitors of Wnt signaling pathway in the field of marine anti-cancer drug development. Our study demonstrated that GTX has therapeutic potential for the prevention or treatment of Wnt dependent cancers and other Wnt related diseases.

## 2. Results

### 2.1. Development of a Cell Based Screen System for Small Molecule Modulators of the Wnt/β-Catenin Pathway

**Figure 1:**
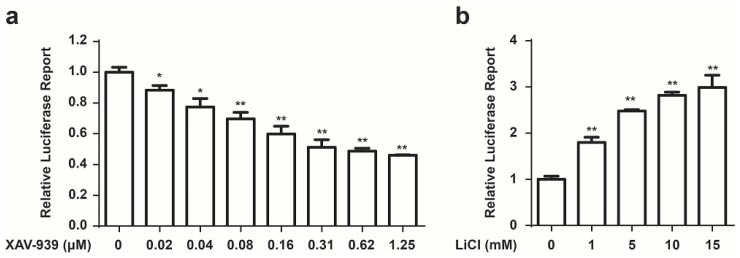
Development of a cell based screen system for small molecule modulators of the Wnt pathway. (**a**) XAV-939 inhibits the Wnt luciferase activity in HCT 116 stable cell line. Cells were treated with indicated concentration of XAV-939 for 24 h, Wnt signaling activity was measured using a Luciferase Assay Kit (Promega, Madison, WI, USA). Data are presented as the mean ± SD (*n* = 3). One-way ANOVA with multiple group comparison by Fisher’s LSD test, * *p* < 0.05, ** *p* < 0.001; (**b**) LiCl increases the Wnt-luciferase activity in HCT 116-Wnt-luc cells. Cells were treated with indicated concentration of LiCl for 24 h, Wnt signaling activity was measured using a Luciferase Assay Kit (Promega, Madison, WI, USA). Mean ± SD (*n* = 3). One-way ANOVA with multiple group comparison by Fisher’s LSD test, ** *p* < 0.001.

We developed a Wnt responsive luciferase reporter assay in HCT 116 cells to identify the small molecule inhibitors of cancer associated constitutive Wnt signaling. HCT 116 cells, which harbor activating mutations of β-catenin, were stably transfected with a luciferase reporter plasmid which contained eight copies of a TCF/LEF response element that drives transcription of the luciferase reporter gene. Luciferase activity was measured to evaluate the modulation of the Wnt/β-catenin transcriptional activity. To increase luciferase assay sensitivity, a single clone with high luciferase expression was chosen to conduct further experiments. Since this system could be used for compound screens, it would be of paramount importance to demonstrate that small molecules were capable of modulating Wnt signaling pathway. Previously, XAV-939, which selectively inhibits β-catenin-mediated transcription, has been identified as an inhibitor of Wnt signaling [20]. Therefore, XAV-939 was used as a positive control compound to determine whether our screen system was effective. As shown in Figure 1a, XAV-939 inhibited luciferase activity in a concentration dependent manner. Meanwhile, we used LiCl to serve as an activator of Wnt signaling [21] to test our system. As shown in Figure 1b, LiCl increased luciferase activity in a concentration dependent manner. These data demonstrated that our stable cell line HCT 116-Wnt-luc could be used for drug screening.

### 2.2. Identification of GTX and Some of Its Analogues as Small Molecule Inhibitors of Wnt Signaling

**Figure 2:**
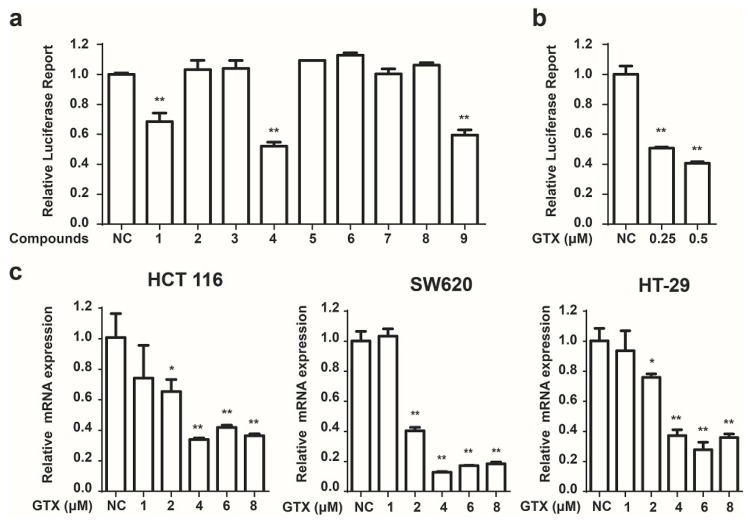
Identification of GTX and some of its analogues as small molecule inhibitors of Wnt signaling. (**a**) Screening of compounds that inhibit the Wnt pathway in HCT 116-Wnt-luc cells. HCT 116-Wnt-luc cells were incubated with 1 μM indicated compounds for 24 h, luciferase activity was measured using Luciferase Assay Kit (Promega, Madison, WI, USA). Mean ± SD (*n* = 3). Student’s *t* test. ** *p* < 0.001; (**b**) GTX inhibits the Wnt-luciferase activity in a concentration dependent manner. HCT 116-Wnt-luc cells were incubated with GTX at indicated concentration for 24 h, luciferase activity was measured using Luciferase Assay Kit (Promega, Madison, WI, USA). Mean ± SD (*n* = 3). One-way ANOVA with multiple group comparison by Fisher’s LSD test, ** *p* < 0.001; (**c**) GTX decreases expression of Wnt target gene c-myc in mRNA level. HCT 116, SW620, HT-29 cells were treated with GTX at various concentrations (1–8 μM) for 24 h, then the expression of c-myc was measured with real-time PCR. GAPDH was used to normalize the relative expression level. Mean ± SD (*n* = 3). One-way ANOVA with multiple group comparison by Fisher’s LSD test, * *p* < 0.05, ** *p* < 0.001.

Next we used these HCT 116-Wnt-luc cells to screen small molecule derived from secondary metabolites of marine fungi (Table 1), which were obtained previously [19]. GTX and some of its analogues, acetylgliotoxin, and reduced gliotoxin were shown strongly to inhibit the luciferase activity (Figure 2a). To identify the specific inhibition to Wnt signaling of molecules, GTX was subsequently tested. As shown in Figure 2b, GTX inhibited the Wnt luciferase activity in a concentration dependent manner. To test whether GTX inhibited the endogenous activity of Wnt signaling, we examined its effect on the downstream of Wnt signaling. Since c-myc was well identified as a target gene of the Wnt signaling pathway in CRC [22], we examined the effect of GTX on c-myc. Real-time PCR assay was performed to detect the mRNA level of c-myc. As shown in Figure 2c, GTX inhibited the expression of endogenous c-myc in three CRC cell lines.

**Table 1:** The secondary metabolites compounds of marine fungi used in these assays

Symbol	Name
1	gliotoxin
2	didehydrobisdethiobis(methylthio)gliotoxin
3	6-acetylbis(methylthio)gliotoxin
4	acetylgliotoxin
5	bisdethiobis(methylthio)gliotoxin
6	bis-*N*-norgliovictin
7	1,2,3,4-tetrahydro-2-methyl-1,3,4-trioxopyrazino[1,2-a]indole
8	1,2,3,4-tetrahydro-2-methyl-3-methylene-1,4-dioxopyrazino[1,2-a]indole
9	reduced gliotoxin

### 2.3. GTX Inhibits Wnt Signaling Pathway by Targetting β-Catenin

**Figure 3:**
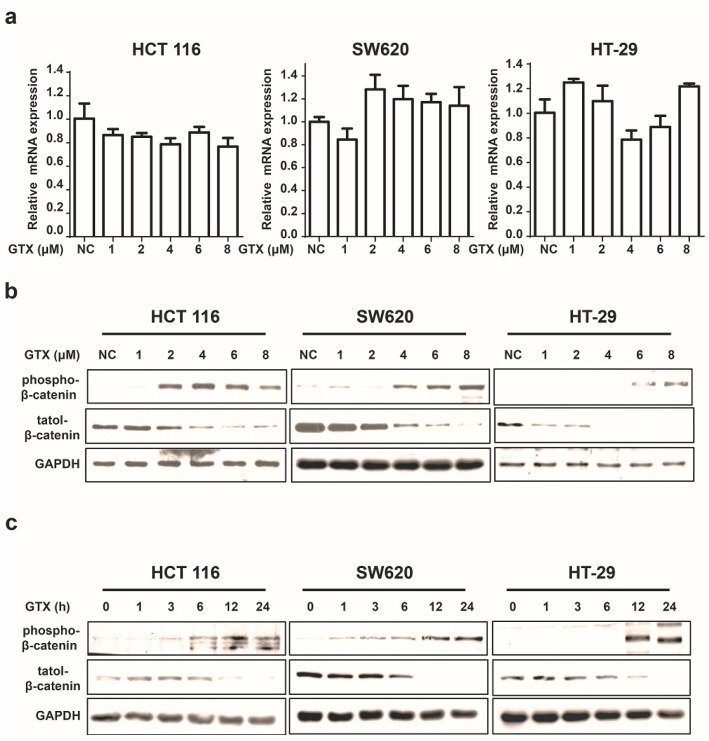
GTX inhibits Wnt signaling pathway by targeting β-catenin. (**a**) GTX has no effect on β-catenin mRNA expression in CRC cells. HCT 116, SW620, HT-29 cells were treated with GTX at various concentrations (1–8 μM) for 24 h. GAPDH was used to normalize the relative expression level. Mean ± SD (*n* = 3). One-way ANOVA with multiple group comparison by Fisher’s LSD test, no significant.; (**b**) GTX increases the phospho-β-catenin level and decreases its total protein level in a concentration dependent manner in CRC cells. HCT 116, SW620, HT-29 cells were treated with GTX at various concentrations (1–8 μM) for 24 h. β-catenin and phospho-β-catenin(Ser33/37) were analyzed by western blot. GAPDH was used as a loading control; (**c**) GTX increases the phospho-β-catenin level and decreases its total protein level in a time dependent manner in CRC cells. HCT 116, SW620, HT-29 cells were treated with 4 μM GTX for indicated time periods (1–24 h). β-Catenin was analyzed by western blot. GAPDH was used as a loading control.

Since β-catenin is a key mediator of the Wnt pathway, we then tested whether GTX inhibited the Wnt pathway by regulating β-catenin. First, we examined its effect on the expression of β-catenin in mRNA levels by real-time PCR assay. As shown in Figure 3a, GTX did not affect the β-catenin mRNA expression. Next, we performed western blot assay to evaluate the protein expression of β-catenin. Treatment of HCT 116, SW620 and HT-29 cells with GTX resulted in a decrease in the protein level of β-catenin in both time and concentration manners (Figure 3b,c). Given that the phosphorylation of Ser33/37/Thre41 residues is prerequisite for β-catenin degradation, we also examined the effect of GTX on the phosphorylation of β-catenin (Ser33/37). As shown in Figure 3b,c, GTX induced β-catenin phosphorylation in both concentration and time dependent manner in HCT 116, SW620 and HT-29 cells. Together these results suggest that GTX antagonizes the Wnt pathway by promoting the degradation of β-catenin protein.

### 2.4. GTX Inhibits Proliferation in CRC Cells

**Figure 4:**
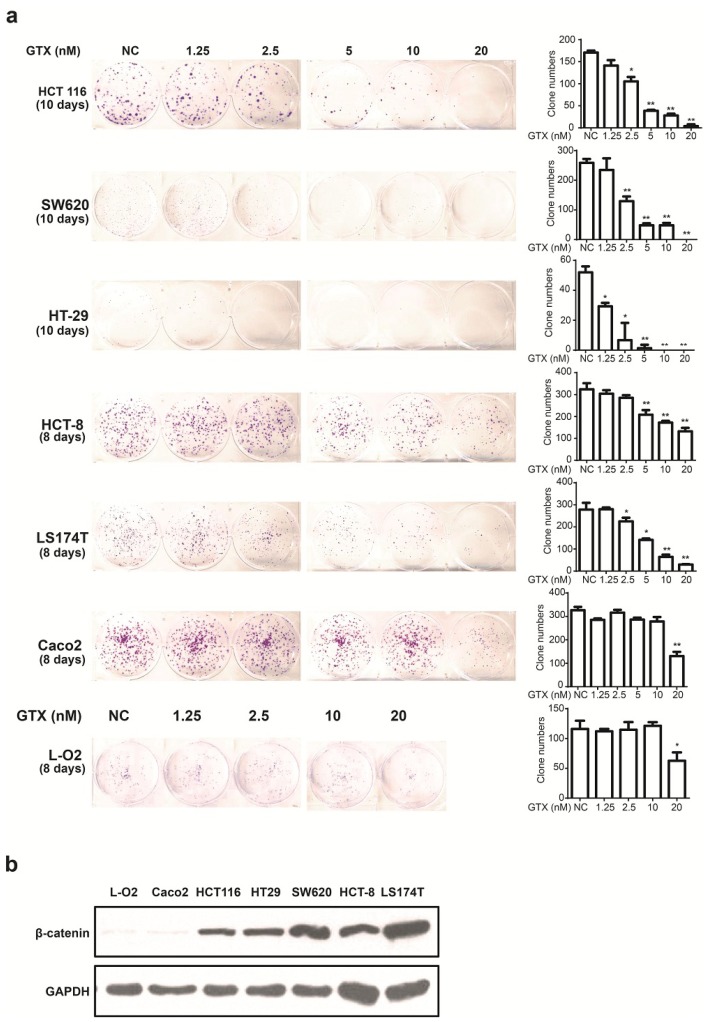
GTX inhibits proliferation of CRC cells. (**a**) (left) Representative images from colony formation assays of CRC cells including HCT 116, SW620, HT-29, HCT-8, Caco-2 LS174T and one non CRC cells L-O2. Cells were treated with indicated concentrations (1.25–20 nM) of GTX for indicated time periods (8–10 days). The cells were then fixed and stained with crystal violet. (right) Representative histograms from colony formation in the six CRC cell lines and one non CRC cell line L-O2 cells. One-way ANOVA with multiple group comparison by Fisher’s LSD test, * *p* < 0.05, ** *p* < 0.001; (**b**) The expression levels of β-catenin from different CRC cell lines and one non CRC cell line were analyzed by western blot analysis. Caco-2 cells expressed significantly lower levels of β-catenin than the other CRC cells. GAPDH was used as a loading control.

Since GTX inhibited Wnt signaling in CRC cells, we examined whether this compound could inhibit the proliferation of six CRC cell lines and one non CRC cell line using the colony formation assay. β-catenin expression levels of a number of cell lines were detected by western blot. As shown in Figure 4b, L-O2 and Caco-2 cells were expressed lower level of β-catenin. As shown in Figure 4a, there was little apparent reduction in cell proliferation in these β-catenin independent cells. This indicated that GTX have a cancer cell selective property and the sensitivity to GTX may result from the expression level of β-catenin.

### 2.5. GTX Induces Apoptosis and Caspase Activation in CRC Cells

**Figure 5:**
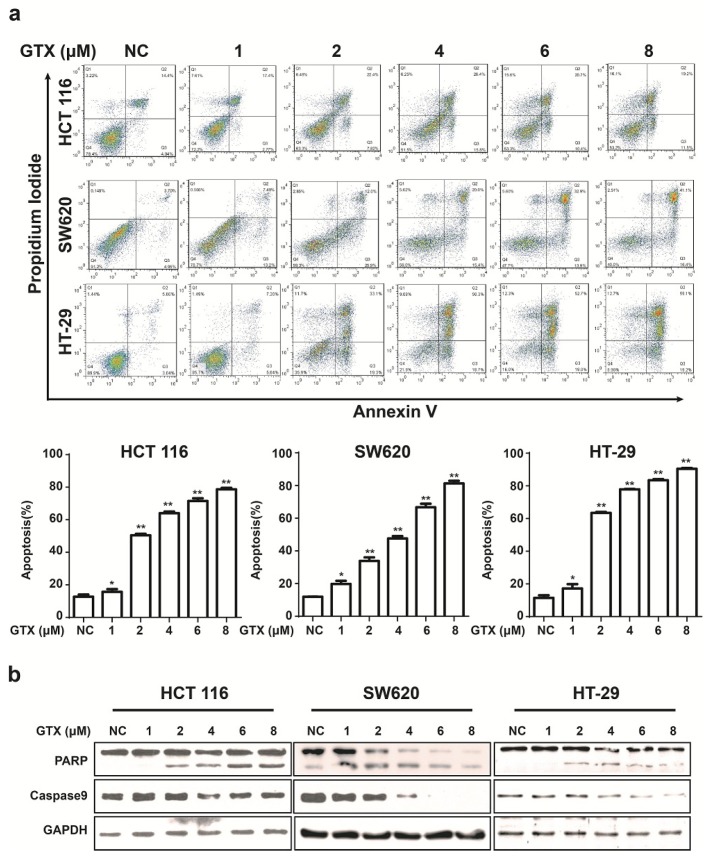
GTX induces apoptosis and caspase activation in CRC cells. (**a**) GTX induces apoptosis in a concentration dependent manner in CRC cells. HCT 116, SW620, HT-29 cells were treated with GTX at various concentrations (1–8 μM) for 24 h, then cells were collected and stained with Annexin V-FITC/PI dual staining (KeyGEN Biotech., Nanjing, Jiangsu, China) for flow cytometric assay. Graphs show data from a representative experiment. Representative histograms from flow cytometric assay in HCT 116, SW620 and HT-29 cells. Mean ± SD (*n* = 3). One-way ANOVA with multiple group comparison by Fisher’s LSD test, * *p* < 0.05, ** *p* < 0.001; (**b**) GTX induces cleavage of PARP and decrease of precursor forms of caspase-9 in CRC cells. HCT 116, SW620, HT-29 cells were treated with GTX at indicated concentrations (1–8 μM) for 24 h, PARP and caspase-9 were analyzed with western blot. GAPDH was used as a loading control.

To examine the ability of GTX to induce apoptosis in Wnt related cancer cells, HCT 116 (harboring activating mutations of β-catenin), HT-29, and SW620 (harboring inactivating mutations of APC mutations) were chosen for further study. Initially, the capacity of GTX to induce cell apoptosis was evaluated by flow cytometry with Annexin V-FITC/PI dual staining. The proportion of apoptotic cells, in concentration dependent manners, increased in CRC cells (Figure 5a). To further verify the induction of apoptosis, apoptosis-associated proteins were measured with western blot assay. As shown in Figure 5b, GTX induced the cleavage of PARP (an indicator of apoptosis) in a concentration dependent manner in CRC cells. Consistently, the levels of the precursor forms of caspase-9 were decreased after GTX treatment, matching the pattern of PARP cleavage. This demonstrated that GTX triggered CRC cells apoptosis via caspase activation.

## 3. Discussion

Marine-derived fungi has been proven to be a promising source of bioactive metabolites. In recent years, we used an alternative cultivation parameters strategy to isolate and identify a series of novel and/or bioactive metabolites from marine fungi [16,17,18,19]. GTX and its analogues, the secondary metabolites of the marine fungus *Neosartorya pseudofischeri*, exerted potential cytotoxic effect on CRC cell lines [19]. To further explore the anti-cancer mechanism of these compounds in CRC cells, we developed a Wnt responsive luciferase reporter system to screen small molecule inhibitors of cancer associated with constitutive activation of the Wnt pathway. In the current study, we showed that a highly sensitive Wnt dependent cell line could be used to identify small-molecule inhibitors of Wnt signaling. The cell based screen described here was similar to previous studies [20,23,24,25] in that it relied on the identification of compounds which blocked the activity of an integrated TCF-luciferase reporter. However, a novel reporter cell line was developed in this study, it has a cancer associated active basal Wnt responsive luciferase reporter and its activity of Wnt promotion or inhibition could be easily regulated by compounds. The advantage of this strategy is that Wnt signaling could be modified without any exogenous stimulation and this cell response to inhibitors or stimulations is commonly found in cancer cell lines. Furthermore, cancer cell lines used in this system could faithfully represent hyper-mutation status of the Wnt pathway, and it has the potential to identify Wnt pathway inhibitors.

GTX, an epipolythiodioxopiperazine metabolite with immunosuppressive properties [26,27,28] has been investigated as an antibiotic and also as an antiviral agent [29]. Recently, it has been reported as a promising anti-cancer agent by inducing apoptosis in human cervical cancer cells, chondrosarcoma cells, lung cancer cells [30], chronic lymphocytic leukemia cells [31], and breast cancer cells [32]. With few exceptions, most tumors are the products of multiple mutations in various aberrant signaling pathways. Consequently, simultaneous targeting inhibition of multiple signaling pathways could be more effective than inhibiting a single pathway in cancer therapies. Compounds with broader targeting specificity may be more effective than reagents with narrower selectivity and may also decrease the likelihood of the development of drug resistance. GTX has been well identified as an NFκB inhibitor [33,34], a potent NOTCH2 transactivation inhibitor [31], and histone methytransferase inhibitor [35]. This is the first study to identify GTX and some of its analogues as potential Wnt pathway inhibitors. Our results show that GTX inhibits the Wnt pathway activity by promoting the degradation of β-catenin. In addition, GTX inhibits the proliferation and induces apoptosis in CRC cells which display high Wnt signaling activity. Other reports have demonstrated the importance of GTX as induction of membrane permeability transition and caspase activation [30,36,37,38]. Consistent with those reports, we showed that GTX-triggered CRC apoptosis is likely via caspase activation. Our results revealed that the potential of GTX and it analogues as inhibitors for Wnt pathway and provide evidence for their anti-cancer activity in CRC cells.

## 4. Experimental Section

### 4.1. Cell Culture, Transfection, and Stable Cell Line Selection

Human epithelial colorectal adenocarcinoma HCT 116, SW620, HT-29, HCT-8, Caco-2 and LS174T cells were purchased from Culture Collection of Chinese Academy of Science (Shanghai, China). Human normal hepatic L-O2 cells were kindly provided by Professor Fang (Sun Yat-sen University, Guangzhou, China). Cells were cultured in RPIM 1640 medium (Gibco Life Technologies, Carlsbad, CA, USA) supplemented with 10% inactivated fetal bovine serum (Gibco Life Technologies, Carlsbad, CA, USA), 100 units/mL penicillin and 10 µg/mL streptomycin (Gibco Life Technologies, Carlsbad, CA, USA) in a humidified atmosphere of 5% CO_2_ at 37 °C.

### 4.2. Reagents

GTX was isolated from the secondary metabolites of marine fungus, *Neosartorya pseudofischeri* by following the procedure described previously [19]. It was dissolved in dimethyl sulphoxide (DMSO), which was from Sigma-Aldrich (St. Louis, MO, USA), to a 50 mM solution and stored at −20 °C. phospho-β-catenin (#2009), PARP (#9542) was purchased from Cell Signaling Technology (Beverly, MA, USA). β-catenin (ab32572), c-myc (ab32072) were purchased from Abcam (Cambridge, MA, USA). GAPDH (10494-1-AP), caspase-9 (66169-1-lg) and anti-rabbit immunoglobulin G horseradish peroxidase-conjugated secondary antibodies were from Proteintech Group (Chicago, IL, USA).

### 4.3. Plasmids and Transfection

Reporter plasmids pRL-TK and pGL4.49 were purchased from Promega (Promega, Madison, WI, USA). The pGL4.49 vector contains eight copies of a TCF-LEF response element (TCF-LEF RE) that drives transcription of the luciferase reporter gene. Cell transfection used lipofectamine3000 (Invitrogen, Grand Island, NY, USA) according to the manufacturer’s instructions. Single transfected colonies were picked in the presence of hygromycin B (Invitrogen, Grand Island, NY, USA).

### 4.4. Luciferase Activity Assay

Luciferase activity assay were performed following the manufacturer’s instructions developed by Promega. In brief, HCT 116 cells stably transfected with pGL4.49 plasmid were seeded and cultured in 24-well plates for 24 h. Cells were incubated with compounds. After 24 h, cell lysate was prepared by employing Luciferase Assay Kit (Promega, Madison, WI, USA) and luciferase activity was measured using a Thermo Scientific Varioskan Flash Multimode Reader (Thermo Fisher Scientific Inc., Waltham, MA, USA).

### 4.5. Colony Formation Assay

Cells were seeded in 6-well plate, with 500 cells per well. The cells were treated with either different concentration of compounds or 0.1% DMSO as vehicle control and cultured in an atmosphere of 5% CO_2_ at 37 °C for the indicated times. Medium was changed every three days. The cells were washed with PBS and fixed in ice-cold methanol for 5 min, and stained with crystal violet. Images of the colonies were photographed. Each treatment was evaluated in triplicates, and representative images were shown.

### 4.6. RNA Analysis and Real-Time PCR

Total RNA was isolated with Trizol reagent (Invitrogen, Grand Island, NY, USA) following the manufacturer’s instructions. The first-strand of cDNA was synthesized from 2 μg of total RNA using the PrimeScript RT reagent Kit (TaKaRa, Dalian, Liaoning, China) and random primers. Real-time PCR was carried out using SYBR Green Premix Ex Taq II Kit (TaKaRa, Dalian, Liaoning, China) in the ABI 7500 system (ABI, New York, NY, USA). Gene expression was normalized to GAPDH and relative quantitation was calculated by using the ΔΔCt method. The specific primers were used as follows: MYC-forward: GGACCCGCTTCTCTGAAAGG, MYC-reverse: TAACGTTGAGGGGCATCGTC, GAPDH-forward: GCACCGTCAAGGCTGAGAAC, GAPDH-reverse: TGGTGAAGACGCCAGTGGA.

### 4.7. Western Blot Analysis

Western blot was performed as previously described [39]. Briefly, cells were lysed in lysis buffer containing protease and phosphatase inhibitors (KeyGEN Biotech., Nanjing, Jiangsu, China). Protein concentrations were measured using a Bio-Rad assay kit (Hercules, CA, USA). Total cellular proteins were separated by SDS-PAGE and transferred to PVDF (Bio-Rad, Hercules, CA, USA) membranes followed by probed with a primary antibody over night at 4 °C. The next day, the membrane was washed and incubated with HRP-conjugated secondary antibody at room temperature for 2 h, followed by ECL (Bio-Rad, Hercules, CA, USA) detection using of a X-ray film or chemiluminescence equipment (ABI, New York, NY, USA). After detection of protein bands, the membrane was stripped and re-probed with anti-GAPDH antibody to confirm equal loading of samples.

### 4.8. Flow Cytometry

For cell apoptosis, cells treated with GTX for the indicated times, were collected, washed with binding buffer, then incubated in working solution (100 μL binding buffer with 0.3 μL Annexin V) for 15 min in the dark. Cells were then washed and resuspended with binding buffer. PI (Sigma-Aldrich, St. Louis, MO) was added just before flow cytometric analysis, apoptotic cells were determined by BD FACScanto II flow cytometry (BD Biosciences, San Jose, CA, USA) and the resulting data were analyzed by BD FACSDiva software version 6.1.3 (BD Biosciences, San Jose, CA, USA).

### 4.9. Statistical Analysis

All experiments were done at least three times. Data were presented as “mean ± SD”. All the data were analyzed by two-tailed unpaired Student’s *t* test between two groups and by one-way ANOVA followed by Fisher’s LSD test for multiple comparison involved. The Statistical analyses were performed using GraphPad Prism software (San Diego, CA, USA). A *p* value of <0.05 was considered to be statistically significant.

## 5. Conclusions

This is the first study to identify GTX and some of its analogues as potential Wnt pathway inhibitors. Our results show that GTX inhibits Wnt pathway activity by promoting the degradation of β-catenin. In addition, GTX inhibits the proliferation and induces apoptosis in CRC cells which display high Wnt signaling activity. Collectively, these results revealed that the potential of GTX and it analogues as inhibitors for Wnt pathway and provide evidence for their anticancer activity in CRC cells.
